# A Method for Assessing the Efficiency of the Nucleotide Excision Repair System Ex Vivo

**DOI:** 10.32607/actanaturae.11430

**Published:** 2021

**Authors:** A. A. Popov, K. E. Orishchenko, K. N. Naumenko, A. N. Evdokimov, I. O. Petruseva, O. I. Lavrik

**Affiliations:** Institute of Chemical Biology and Fundamental Medicine SB RAS, Novosibirsk, 630090 Russia; Institute of Cytology and Genetics SB RAS, Novosibirsk, 630090 Russia; Novosibirsk National Research State University, Novosibirsk, 630090 Russia

**Keywords:** nucleotide excision repair, ex vivo methods, DNA damages

## Abstract

The nucleotide excision repair (NER) is one of the main repair systems present in the cells of living organisms. It is responsible for the removal of a wide range of bulky DNA lesions. We succeeded in developing a method for assessing the efficiency of NER in the cell (*ex vivo*), which is a method based on the recovery of TagRFP fluorescent protein production through repair of the damage that blocks the expression of the appropriate gene. Our constructed plasmids containing bulky nFlu or nAnt lesions near the *tagrfp *gene promoter were shown to undergo repair in eukaryotic cells (HEK 293T) and that they can be used to analyze the efficiency of NER *ex vivo*. A comparative analysis of the time dependence of fluorescent cells accumulation after transfection with nFlu- and nAnt-DNA revealed that there are differences in how efficient their repair by the NER system of HEK 293T cells can be. The method can be used to assess the cell repair status and the repair efficiency of different structural damages.

## INTRODUCTION

The nucleotide excision repair (NER) system removes the bulky DNA lesions resulting from exposure to various factors: chemically active compounds, UV, and X-ray. There are two types of NER. Global genome NER is responsible for the search and removal of bulky lesions in the entire genome, regardless of its functional state, using XPC factor complexes for primary recognition of the damage site [1]. Transcription-coupled NER is activated by stalling of the RNA polymerase II transcription complex by a bulky lesion in the transcribed DNA strand [2]. About 30 protein factors and enzymes, identical in both NER types, then form a number of complexes on DNA which perform lesion removal, repair synthesis, and ligation.

The use of approaches that focus on exploring the structure and functions of the proteins involved in NER has the potential to help elucidate the process mechanism and to identify the main stages affecting its efficiency, as well as the composition and structure of the multiprotein complexes that appear and act at different NER stages [1, 3]. In most studies, the activity of the eukaryotic NER system *in vitro *is assessed using extended DNA containing natural bulky lesions at a given position or their synthetic analogs, as well as fractionated cell extracts containing a set of NER proteins (NER-competent extracts) [4, 5]. Nevertheless, the development of approaches that can help investigate and compare efficiency in bulky lesion repair in living cells (*ex vivo*) remains topical in both fundamental and applied research.

**Fig. 1:**
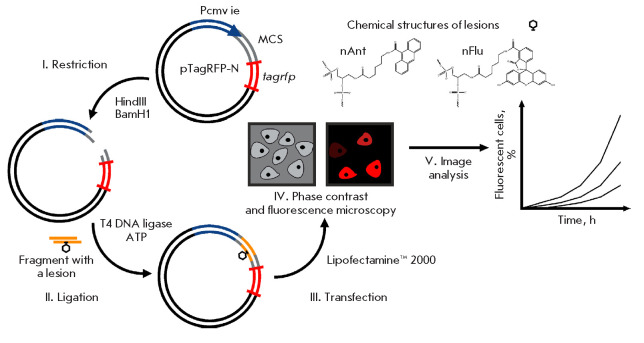
Schematic of a method for assessing the NER system efficiency *ex vivo*

paper describes a method for such assessments using model plasmids with a bulky lesion near the promoter region of the gene encoding the TagRFP fluorescent protein. The schematic for creating model plasmids with a bulky lesion and assessing the efficiency of NER *ex vivo *through monitoring of the recovery of reporter fluorescent protein expression, which happened to be impaired by a bulky DNA lesion, by the repair machinery of eukaryotic cells is shown in *Fig. 1*.

## EXPERIMENTAL

HEK 293T cells were cultured in a IMDM medium (Gibco) supplemented with 10% FBS (Gibco), 1% GlutaMAX™ Supplement (Gibco), 105 U/L penicillin, 100 mg/L streptomycin, and 2.5 mg/L amphotericin β at 37°C and 5% CO_2_.

ODNs for creating inserts were synthesized in the Laboratory of Biomedical Chemistry (Institute of Chemical Biology and Fundamental Medicine SB RAS) according to the procedure described in [5]. The ODN sequences are shown in the *Table*.

**Table:** ODN sequences

No.	ODN
1	5’-P-agctgctgctcatctcgagatctgagtacattggattgccattctccgagtgtattaccgtgacg-3’
2	5’-P-gatccgtcacggtaatacactcggagaatggcaatccaatM1tactcagatctcgagatgagcagc-3’, where M1 is nFlu
3	5’-P-gatccgtcacggtaatacactcggagaatggcaatccaatM2tactcagatctcgagatgagcagc-3’, where M2 is nAnt

A 38-bp segment (622–660 bp, MCS) was excised from the pTagRFP-N plasmid using the restriction endonucleases HindIII and BamHI (SibEnzyme) by incubation of 1 μg of the plasmid with 1 U HindIII and 1 U BamHI in a W buffer (SibEnzyme) at 37°C for 1 h. After enzyme inactivation (70°C, 20 min) and DNA precipitation according to the standard procedure [6], the linearized plasmid was dissolved in water and a 40-fold molar excess of the DNA insert, 2 U T4 DNA ligase (SibEnzyme) in a SE buffer, and 5 mM ATP were added. The plasmid was ligated at 12°C for 16 h. Then, the reaction mixture was warmed up (65°C, 20 min) and the DNA from the reaction mixture after ligation was separated in 0.8% agarose gel. The circular plasmid with inserts was eluted from the agarose gel using a DNA elution kit (diaGene), according to the manufacturer’s protocol.

Transfection of cells with the plasmid was performed using Lipofectamine™ 2000 (Invitrogen), according to the manufacturer’s protocol. The cells were seeded onto a 24-well plate at an amount of 2.5 × 10^4^ cells per well in 500 μL of a culture medium containing no antibiotics. Upon reaching 50–0% confluence, the medium was removed and the cells were added with a complex of the plasmid (150 ng) and the Lipofectamine ™2000 reagent in a serum-free medium. Fluorescence was detected using the Cell-IQ MLF system (Chip-Man Technologies, Finland) for longterm intravital monitoring of the cells at the Common Use Center of the Institute of Cytology and Genetics, SB RAS. The cells were pictured at 10-min intervals in the phase contrast and fluorescence modes using a Nikon CFI Plan Fluorescence DL ×10 objective. The resulting images were analyzed using the ImageJ and Cell-IQ Analyzer software.

The statistical analysis was performed using the Statistica10 software. All experiments were performed at least in triplicate, and the statistical significance was determined using the Student’s *t*-test.

## RESULTS AND DISCUSSION

The approach based on the reactivation of the fluorescent protein expression after removal of a DNA lesion that blocks the expression has been successfully used in NER studies [7, 8]. We decided to modify this approach in order to detect the fluorescence signal in living cells using the Cell-IQ MLF device for intravital examination, which combines a microscope with phase contrast and fluorescence imaging modes, as well as a system for supplying CO_2_ and maintaining temperature, ensuring optimal conditions for the cells during a prolonged imaging process. The software supplied with the device enables one to analyze images and extract information on the total number and the number of cells expressing fluorescent proteins, the fluorescent signal intensity, cell motility, and other parameters.

To create DNA with bulky lesions, we used the pTagRFP-N vector (4.7 kbp) containing the *tagrfp* gene encoding the monomeric fluorescent protein RFP from the sea anemone *Entacmaea quadricolor *[9]. The advantages of using TagRFP include the generated bright fluorescent signal, the stability of the protein at high pHs, rapid maturation, and the absence of toxic effects on the cells. The *tagrfp* gene is driven by the early promoter of cytomegalovirus (Pcmv ie), which is adjacent to a multiple-cloning site (MCS) with recognition sites for various restriction endonucleases, which enables cloning of the required DNA insert into this region.

Recombinant plasmids containing bulky nFlu and nAnt lesions (hereinafter referred to as nFlu and nAnt DNA, respectively) were synthesized. The pronounced substrate properties of these lesions, which were revealed in a specific excision reaction catalyzed by NER proteins from various cell extracts *in vitro *[5, 10], were taken into account when using nFlu and nAnt to create model plasmids.

**Fig. 2:**
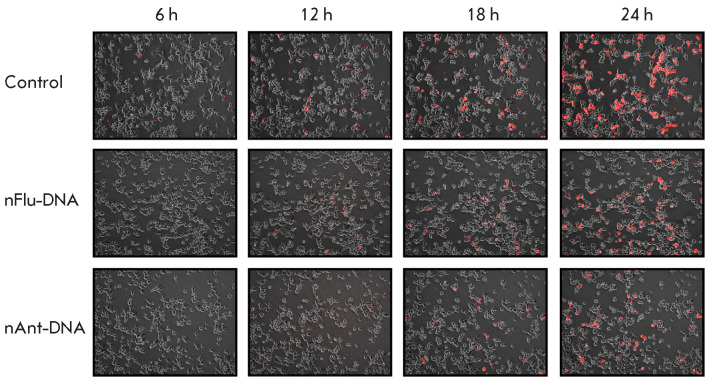
TagRFP expression in HEK 293T cells transfected with plasmid DNAs. The images were created by overlay of fluorescence and phase-contrast images in ImageJ. Plasmid DNA substrates are shown on* left*; time after cell transfection is shown on *top*

The efficiency in NER of nFlu- and nAnt-DNA in HEK 293T human embryonic kidney cells was analyzed. We assessed the time of emergence of cells whose fluorescence indicated recovery of the TagRFP protein expression (*Fig. 2*). A plasmid with a DNA insert without a bulky lesion was used as a control. An evaluation of the number of fluorescent cells in the total cell population using the Cell-IQ Analyzer and ImageJ revealed differences in efficiency between the nAntand nFlu-DNA repair systems. In nAnt-DNA-transfected cells, the first fluorescent cells were detected 10 h after transfection, while in nFlu-DNA-transfected cells, the first fluorescent cells were observed after 8 h (*Fig. 3A*). The number of fluorescent cells 12 h after transfection was 1.56 Ѓ} 0.39% in the case of nAnt-DNA-transfected cells and 4.59 Ѓ} 0.76% in the case of nFlu-DNA-transfected cells (*Fig. 3B*). To achieve a similar number of fluorescent cells transfected with nAnt-DNA, it took another 2 h, and the number was 4.27 Ѓ} 0.67% after 14 h.

**Fig. 3:**
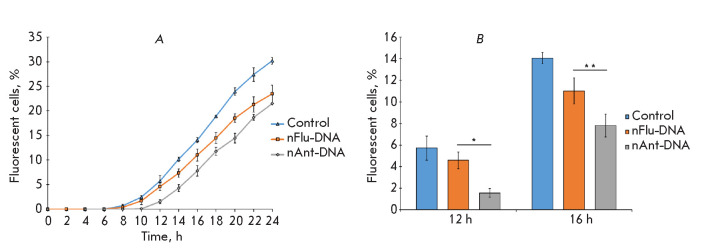
3. Analysis of the NER efficiency of plasmid DNAs *ex vivo *in HEK 293T cells. (*A*) – the number of fluorescent cells (%) over time after transfection with plasmid DNAs; (*B*) – a representative diagram demonstrating the differences in the quantities of fluorescent cells transfected with nFlu- or nAnt-DNA 12 h and 16 h after transfection. The confidence levels are *p  < 0.01 and **p  < 0.05

The repair of nFlu-DNA proceeds faster than the repair of nAnt-DNA, which is consistent with the results observed for the repair of the nAnt- and nFlu-DNA duplexes *in vitro *in the presence of proteins of NER-competent extracts from various cancer cell lines (HeLa, SiHa, C33A) [5].

Many factors underlie the difference in the efficiency of bulky lesion repair when using the NER system. These may be the structural damage differences that determine the nature of the primary recognition of the damaged site and the efficiency of the subsequent verification of the damage by the proteins of the TFIIH complex [11], as well as the rate and efficiency of a NER system response in various cells to the damaging effect. Further investigation of NER using a combination of *in vitro *and *ex vivo *approaches may enduce significant progress in our understanding of this process in eukaryotic cells.

## CONCLUSIONS

Therefore, the proposed method enables one to assess efficiency in the removal of bulky nAnt and nFlu lesions from model plasmids by the NER system of HEK 293T cells. The method is a promising tool for studying NER; it enables one to compare both the repair status of various cells and efficiency in the repair of various structural lesions.

## Abbreviations

NER: nucleotide excision repair
ODN: oligodeoxyribonucleotide
ATP: adenosine triphosphate
nFlu: N-[6-(5(6)-fluoresceinylcarbamoyl)hexanoyl]-3-amino-1,2-propandiol
nAnt: N-[6-(9-antracenylcarbamoyl) hexanoyl]-3-amino-1,2-propandiol
MCS: multiple cloning site
kbp: kilo base pairs
